# Blood Biomarkers of Alzheimer's Disease and Patterns of Structural Brain Changes in the Community

**DOI:** 10.1002/ana.78309

**Published:** 2026-07-27

**Authors:** Martina Valletta, Davide Liborio Vetrano, Erika J. Laukka, Grégoria Kalpouzos, Javier Oltra, Debora Rizzuto, Marco Canevelli, Claudia Fredolini, Chengxuan Qiu, Bengt Winblad, Laura Fratiglioni, Giulia Grande

**Affiliations:** ^1^ Aging Research Center, Department of Neurobiology, Care Sciences and Society Karolinska Institutet and Stockholm University Stockholm Sweden; ^2^ Stockholm Gerontology Research Center Stockholm Sweden; ^3^ Division of Clinical Geriatrics, Department of Neurobiology, Care Sciences and Society Karolinska Institutet Stockholm Sweden; ^4^ Department of Human Neuroscience Sapienza University Rome Italy; ^5^ Affinity Proteomics Stockholm, Science for Life Laboratory, Department of Protein Science, School of Engineering Sciences in Chemistry, Biotechnology and Health (CBH) Royal Institute of Technology (KTH) Solna Sweden; ^6^ Division of Neurogeriatrics, Department of Neurobiology, Care Sciences and Society Karolinska Institutet Solna Sweden; ^7^ Theme Inflammation and Aging Karolinska University Hospital Huddinge Sweden

## Abstract

**Objective:**

We aimed to investigate the associations between Alzheimer's disease (AD)‐related blood biomarkers and changes in brain volumes and cerebrovascular burden in community‐dwelling older adults.

**Methods:**

We included 361 dementia‐free participants with a Mini‐Mental State Examination (MMSE) score ≥ 27 and without prior cerebrovascular events from a Swedish population‐based study. Blood phosphorylated‐tau217 (p‐tau217), neurofilament light chain (NfL), and glial fibrillary acidic protein (GFAP) were measured at baseline. Brain magnetic resonance imaging (MRI) was performed at baseline and after 3 and/or 6 years. Linear mixed models were used to examine associations between AD‐related blood biomarkers and changes in volumes of total brain tissue (TBTV), lateral ventricles, hippocampus, amygdala, and white matter hyperintensities (WMHs).

**Results:**

During the follow‐up, higher p‐tau217 was associated with faster volume loss in the hippocampus (β*year = −0.047, 95% confidence interval [CI] = −0.077 to −0.018) and in the amygdala (β*year = −0.064, 95% CI = −0.105 to −0.022). Elevated GFAP was linked to faster TBTV decline (β*year = −0.040, 95% CI = −0.059 to −0.021), ventricular enlargement (β*year = 0.028, 95% CI = 0.013 to 0.042), and hippocampal shrinkage (β*year = −0.038, 95% CI = −0.064 to −0.013). Participants with higher NfL showed steeper TBTV decline, ventricular enlargement, hippocampal and amygdala shrinkage, and faster WMH accumulation. Results remained largely consistent after excluding participants with mild cognitive impairment at baseline.

**Interpretation:**

In cognitively unimpaired older adults, elevated blood p‐tau217 was linked to faster shrinkage in AD‐specific brain regions, whereas NfL and GFAP were associated with more widespread atrophy, with NfL also associated with accelerated WMH accumulation. These findings suggest that AD‐related blood biomarkers may capture different dementia‐related structural brain changes already at early stages. ANN NEUROL 2026;100:987–997

Blood biomarkers indicating Alzheimer's disease (AD) pathology and related neuropathological processes are rapidly emerging as promising tools for dementia diagnosis. Among these, phosphorylated tau217 (p‐tau217) has emerged as an accurate marker of AD pathology, offering a minimally invasive and cost‐effective alternative to positron emission tomography and cerebrospinal fluid (CSF) measures.^1^, ^2^, ^3^ By contrast, neurofilament light chain (NfL) and glial fibrillary acidic protein (GFAP) reflect less AD‐specific neuropathological alterations, including neuronal injury and astroglial activation.^2^ Although their levels may rise in response to AD pathology, they are less disease‐specific and can also be elevated in other neurodegenerative or cerebrovascular conditions.^2^, ^3^

Elevated concentrations of blood p‐tau isoforms, NfL, and GFAP, have been consistently associated with faster cognitive decline and increased risk of developing dementia.^4^, ^5^, ^6^, ^7^ Whereas evidence derives mostly from clinical cohorts and specialized settings,^8^, ^9^, ^10^ recent studies, including work from our group, suggest that their relevance extends to community‐based populations, where elevated levels of AD‐related blood biomarkers can precede a clinical diagnosis of dementia by up to a decade.^4^, ^5^, ^6^, ^7^, ^11^ Nevertheless, whether these biomarkers are linked with structural brain alterations, and their progression over time, in cognitively unimpaired older adults living in the community remains an open question.^12^

Structural brain changes typically develop years before the clinical onset of dementia. Magnetic resonance imaging (MRI) provides a noninvasive assessment of brain structure, enabling the detection of subtle alterations well before clinical symptoms become evident. As highlighted in a recent systematic review,^12^ a growing number of studies have reported associations between AD‐related blood biomarkers and structural brain alterations on MRI, including both brain atrophy and, to a lesser extent, vascular damage. However, most of the studies had a cross‐sectional design^13^, ^14^ and were conducted in specialized clinical settings, enrolling individuals referred to memory clinics and presenting with a broad spectrum of cognitive symptoms, including overt dementia. Establishing whether AD‐related blood biomarkers are linked with brain alterations in older adults before any clinical diagnosis of dementia, is essential to advance our understanding of the brain correlates of these biomarkers from the earliest disease stage. In addition, to date, research has predominantly focused on NfL; previous findings from clinical cohorts showed that individuals with higher NfL levels have greater brain atrophy^15^, ^16^, ^17^ and, in some cases, more pronounced alterations in white matter integrity and larger white matter hyperintensities (WMHs)^17^, ^18^, ^19^ than those with lower levels. By contrast, evidence on other AD‐related blood biomarkers remains limited, particularly for p‐tau217, which has recently emerged as the most specific and reliable blood biomarker for AD detection.^20^

This study aimed to investigate the associations between AD‐related blood biomarkers – including p‐tau217, NfL, and GFAP – and changes in MRI markers of global and regional brain atrophy and cerebrovascular burden (WMH volume) in a population‐based cohort of cognitively unimpaired older adults.

## Methods

We analyzed data from the MRI sub‐study of the Swedish National study on Aging and Care in Kungsholmen (SNAC‐K‐MRI). SNAC‐K is an ongoing longitudinal, population‐based study that began in 2001.^21^ At baseline (2001–2004), 3,363 randomly selected residents of the Kungsholmen district in Stockholm, all aged 60 years or older, were enrolled. Among SNAC‐K participants, a subgroup of 555 dementia‐free, non‐disabled, individuals underwent structural brain MRI scans at 1.5 Tesla. Follow‐up scans were performed after 6 years in the younger cohorts (<78 years) and after both 3 and 6 years in the older cohorts (≥78 years). At baseline, we excluded 32 participants with prior cerebrovascular events (ie, transient ischemic attack, ischemic or hemorrhagic stroke, or intracranial hemorrhage), 6 with arachnoid cyst, 4 with meningioma, 19 with other neurological diseases, and 16 with a Mini Mental State Examination (MMSE) score < 27. Among the remaining participants, 45 had suboptimal MRI quality and 72 had missing data in at least one AD‐related blood biomarker, leaving a baseline population of 361 participants (Supplementary Fig S1). Participants with missing data in blood biomarkers were, on average, older (mean difference = 2.96, 95% confidence interval [CI] = 0.78–5.14 years) and had a higher number of chronic diseases (mean difference = 0.61, 95% CI = 0.10–1.13) at baseline than those with complete biomarkers data.

The protocol for all waves of the SNAC‐K study was approved by the Regional Ethical Review Board in Stockholm (Dnrs: KI 01‐114, 04‐929/3, Ö26‐2007, 2009/595‐32, and 2023‐02375‐02). All participants provided written informed consent to participate at each study visit, and ethical standards of the Declaration of Helsinki were followed throughout the investigation.

The study was reported following the Strengthening the Reporting of Observational Studies in Epidemiology (STROBE) recommendations.^22^

### Data Collection

At each study visit, data were collected by trained nurses and physicians following standardized procedures. Sociodemographic information (age, sex, and education) was obtained through face‐to‐face interviews. Education was categorized as elementary, high school, or university or higher. Information on chronic diseases was obtained by physicians through clinical evaluation, medical records, self‐reports, laboratory tests, and medication use. Diagnoses were coded according to the International Classification of Diseases 10th revision (ICD‐10).^23^ Body mass index (BMI) was calculated as weight in kilograms divided by height in meters squared (kg/m^2^). *APOE* genotyping was conducted on DNA extracted from peripheral blood samples and participants were classified as *APOE ε*4 carriers if they had at least one *ε*4 allele, or as non‐carriers otherwise.

### Cognitive Assessment and Dementia Diagnosis

The MMSE, administered by the examining physician, was used as a measure of global cognition. Dementia was clinically diagnosed at each wave using the Diagnostic and Statistical Manual of Mental Disorders, Fourth Edition (DSM‐IV) criteria.^24^ The diagnostic procedure involved 3 steps: an initial assessment by the examining physician, a second review by another physician, and, if discordant, a final decision by a senior neurologist. For participants who died between follow‐ups, information from medical records, clinical charts, and the Swedish National Cause of Death Register was used for dementia diagnosis. Mild cognitive impairment (MCI) was defined based on performance on a neuropsychological battery assessing 5 cognitive domains. Test scores were standardized into age‐specific z‐scores, and MCI was defined as performance ≤1.5 SD below the mean in at least one domain, in the absence of dementia and with preserved functional independence.^11^

### Alzheimer's Disease‐Related Blood Biomarkers

Peripheral venous blood samples were collected at baseline, and serum aliquots were stored at −80°C in cryogenic vials at the Karolinska Institutet Biobank. Biomarker analyses were conducted at the Affinity Proteomics Stockholm Unit (SciLifeLab). The following Simoa kits were used: ALZpath pTau‐217 Advantage PLUS for serum p‐tau217; and Neuro 2‐plex B for serum NfL and GFAP. One p‐tau217 measurement below the limit of detection was replaced with a value of 0. Blood biomarkers were dichotomized (high/low) using cutoffs derived from a previous study conducted in the same cohort,^4^ where optimal cutoffs were derived in a training dataset (80%) to predict 10‐year all‐cause dementia and validated in a testing dataset (20%).

### Neuroimaging Data Acquisition and Processing

All participants underwent 1.5T MRI scans (Philips Intera, The Netherlands) using the same scanner and sequences at baseline and follow‐ups. Information on the data acquisition protocol is detailed in the Supplementary Methods.

The volumes of total brain tissue (TBTV) and lateral ventricles were used as indicators of global atrophy, whereas hippocampus and amygdala volumes were considered markers of regional AD‐related atrophy, as these regions are among the earliest to show volume loss in AD.^25^, ^26^ Using SPM12 in MATLAB, T1‐weighted images were segmented into grey matter volume (GMV), white matter (WMV), and cerebrospinal fluid volume (CSFV). TBTV was defined as the sum of GMV and WMV, and total intracranial volume (TIV) as the sum of GMV, WMV, and CSFV. The volumes of hippocampus and amygdala were derived from FreeSurfer automated segmentation and calculated as the sum of left and right hippocampus/amygdala. Ventricular volumes were obtained with the ALVIN toolbox. In addition to the abovementioned volumes, we also calculated the medial temporal atrophy (MTA) score, which was derived using Automatic Visual Ratings of Atrophy (AVRA).^27^ AVRA is an artificial intelligence (AI)‐based tool that automatically rates medial temporal atrophy according to the Scheltens’ scale, a 0 to 4 visual rating scale where higher scores indicate greater atrophy.^28^

WMHs were assessed as a marker of cerebral microvascular pathology.^29^ A senior neuroimaging analyst (author G.K.) manually delineated WMHs on fluid‐attenuated inversion recovery (FLAIR) images.

All volumetric measures were adjusted for TIV using the residual method,^30^, ^31^ and standardized into z‐scores based on baseline population mean and SD to facilitate comparison across coefficients.

### Statistical Analysis

Linear mixed models were used to estimate the association between the levels of AD‐related blood biomarkers at baseline and the levels (intercept) and rates of change (slope) in TBTV, lateral ventricles, hippocampus and amygdala volumes, MTA score, and WMHs volume. All models included random participant‐specific intercepts and random slopes for follow‐up time. The follow‐up time*biomarker interaction term was included as a fixed effect.

All analyses were adjusted for potential confounders including age, sex, education, heart disease, chronic kidney disease, BMI, and anemia.^32^ Additional adjustments included *APOE ε*4 carrier status. As a sensitivity analysis, biomarkers were also modeled as continuous variables.

To further restrict the analyses to a cognitively unimpaired population, all the analyses were repeated after the exclusion of participants with MCI at baseline (N = 68) or of those who developed dementia during the 6‐year MRI follow‐up (N = 13).

Statistical analyses were conducted using R software version 4.3.1 (The R Foundation for Statistical Computing, Vienna, Austria).

## Results

The 361 participants had a mean (SD) age of 69.7 (8.5) years; 58.2% were women, and 41.0% had a university‐level education or higher. The mean (SD) MMSE score of the study population was 29.2 (SD = 0.8). At baseline, individuals with high levels of AD‐related blood biomarkers were older, had lower educational attainment, and showed a greater burden of chronic diseases compared with those with low biomarker levels. They also had smaller TBTV, larger ventricular volumes, smaller volumes of amygdala and hippocampus, and larger WMH volume. Baseline characteristics of study participants, overall and according to baseline levels of p‐tau217, NfL, and GFAP, are reported in the Table 1. Individual trajectories of brain volumes, stratified by baseline levels of p‐tau217, NfL, and GFAP are shown in Supplementary Figures S2 and S3. The distribution of participants across high and low biomarker levels is presented in Supplementary Table S1.

**TABLE 1:** Baseline Characteristics of the Study Population, Overall and by Levels of p‐tau217, NfL, and GFAP

Characteristic	Overall N = 361	Low p‐tau217 N = 269	High p‐tau217 N = 92	Low NfL N = 255	High NfL N = 106	Low GFAP N = 250	High GFAP N = 111
Demographics							
Age, yr	69.7 (8.5)	68.2 (7.8)	74.2 (8.7)	66.8 (6.8)	76.8 (7.8)	67.3 (7.4)	75.1 (8.2)
Sex, F	210 (58.2%)	165 (61.3%)	45 (48.9%)	148 (58.0%)	62 (58.5%)	137 (54.8%)	73 (65.8%)
Education, university	148 (41.0%)	121 (45.0%)	27 (29.3%)	120 (47.1%)	28 (26.4%)	118 (47.2%)	30 (27.0%)
MMSE score	29.2 (0.8)	29.3 (0.8)	29.2 (0.9)	29.3 (0.8)	29.0 (0.9)	29.3 (0.8)	29.1 (0.9)
APOE, at least one *ε*4 allele	98 (27.2%)	67 (24.9%)	31 (33.7%)	67 (26.3%)	31 (29.3%)	68 (27.2%)	30 (27.0%)
Chronic diseases							
Chronic kidney disease	90 (24.9%)	61 (22.7%)	29 (31.5%)	34 (13.3%)	56 (52.8%)	39 (15.6%)	51 (45.9%)
Heart disease	54 (15.0%)	30 (11.2%)	24 (26.1%)	28 (11.0%)	26 (24.5%)	38 (15.2%)	16 (14.4%)
Anemia	25 (6.9%)	14 (5.2%)	11 (12.0%)	13 (5.1%)	12 (11.3%)	16 (6.4%)	9 (8.1%)
BMI, kg/m^2^	26.1 (3.9)	26.1 (4.0)	26.1 (3.7)	26.2 (4.1)	25.7 (3.5)	26.1 (4.0)	26.1 (3.7)
Structural MRI measures							
TBTV, ml	1,068.4 (72.6)	1,078.0 (67.6)	1,040.2 (79.4)	1,088.1 (61.7)	1,021.1 (75.4)	1,084.0 (65.3)	1,033.4 (76.3)
Ventricular volume, ml	37.9 (16.3)	35.7 (14.9)	44.3 (18.6)	34.4 (14.7)	46.5 (17.0)	34.4 (14.5)	45.8 (17.5)
Hippocampus volume, ml	7.6 (0.8)	7.7 (0.7)	7.3 (1.0)	7.8 (0.7)	7.1 (0.9)	7.7 (0.7)	7.1 (0.8)
Amygdala volume, ml	2.7 (0.3)	2.8 (0.3)	2.7 (0.3)	2.8 (0.3)	2.6 (0.4)	2.8 (0.3)	2.6 (0.3)
WMH volume, ml	5.4 (8.9)	4.5 (7.8)	7.8 (11.2)	3.8 (7.0)	9.1 (11.4)	4.3 (7.2)	7.8 (11.4)

Continuous variables are reported as mean (SD), categorical variables are reported as n (%). Blood biomarkers were dichotomized (high/low) using previously derived optimal cutoffs for 10‐year all‐cause dementia prediction. Missing: 7 in *APOE*, 11 in hippocampus, 11 in amygdala volume; and 4 in WMHs volume.

*APOE* = apolipoprotein E; BMI = body mass index; GFAP = glial fibrillary acidic protein; MMSE = mini mental state examination; MRI = magnetic resonance imaging; NfL = neurofilament light chain; p‐tau217 = phosphorylated tau 217; TBTV = total brain tissue volume; WMH = white matter hyperintensity.

Participants underwent an average of 1.6 MRI scans over 5.5 years. During the follow‐up 13 participants developed dementia. Overall, 133 participants died or dropped out before the first follow‐up MRI, and an additional 6 died or dropped out after the first follow‐up. Those who did not complete the follow‐up were, on average, older (mean difference = 2.13, 95% CI = 0.32–3.95 years) and had a greater number of chronic diseases (mean difference = 0.48, 95% CI = 0.04–0.92) at baseline than those who completed the follow‐up. They also had higher levels of p‐tau217 and NfL and lower TBTV. The flow chart of study participation is reported in Supplementary Figure S1.

### Associations Between AD‐Related Blood Biomarkers and MRI Markers of Neurodegeneration

We examined the associations between baseline levels of p‐tau217, NfL, and GFAP, dichotomized using previously derived cutoffs,^4^ and both baseline standardized brain volumes, as well as their rate of change during follow‐up, as shown in Figure 1 and in Table S2.

**FIGURE 1:**
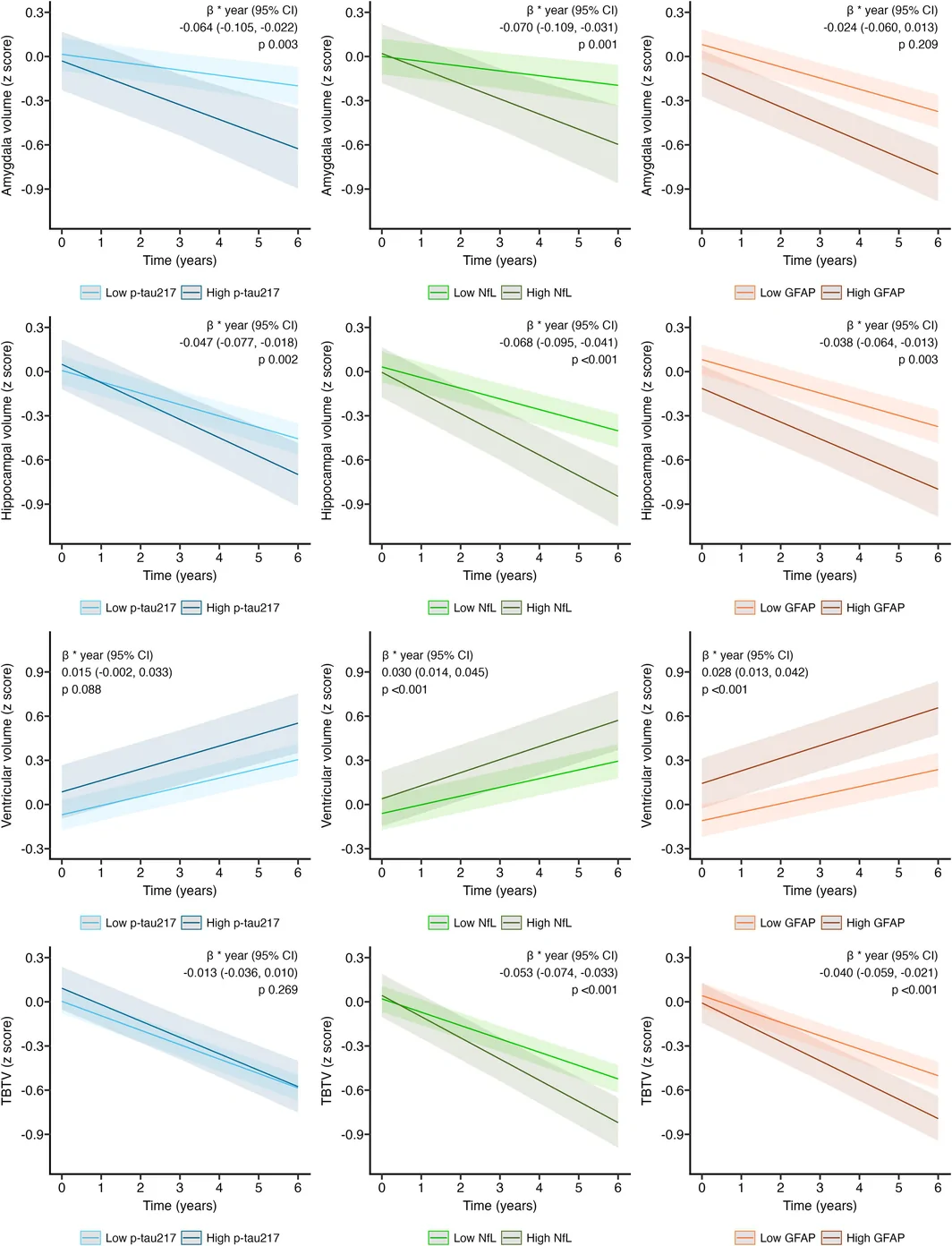
Annual rate of change in volumes of amygdala, hippocampus, lateral ventricles, and TBTV, by baseline levels of p‐tau217, NfL, and GFAP. Linear mixed models adjusted for age, sex, education, chronic kidney disease, anemia, heart disease, and BMI. All volumes were adjusted for total intracranial volume. Blood biomarkers were dichotomized (high/low) using previously derived optimal cutoffs for 10‐year all‐cause dementia prediction. Cutoffs: 0.134 pg/ml for p‐tau217, 20.171 pg/ml for NfL, and 142.515 pg/ml for GFAP. Number of participants: 361 at baseline, 46 at the 3‐year follow‐up, and 185 at the 6‐year follow‐up. BMI = body mass index; GFAP = glial fibrillary acidic protein; NfL = neurofilament light chain; p‐tau217 = phosphorylated tau 217; TBTV = total brain tissue volume.

At baseline, higher GFAP concentrations were linked with larger ventricular volume (*β* = 0.253, 95% CI = 0.042–0.464) and smaller hippocampal volume (*β* = −0.196, 95% CI = −0.392 to 0.000; see Supplementary Table S2).

During the follow‐up (see Fig 1), participants with high levels of p‐tau217 showed faster volume loss in the hippocampus (additional annual change [β*year] = −0.047, 95% CI = −0.077 to −0.018) and in the amygdala (β*year = −0.064, 95% CI = −0.105 to −0.022), compared with those with low levels. Elevated GFAP levels were linked with steeper decline in TBTV (β*year = −0.040, 95% CI = −0.059 to −0.021), faster ventricular enlargement (β*year = 0.028, 95% CI = 0.013–0.042) and faster hippocampal volume loss (β*year = −0.038, 95% CI = −0.064 to −0.013). Participants with high NfL levels exhibited all these changes, showing steeper decline in TBTV (β*year = −0.053, 95% CI = −0.074 to −0.033), faster ventricular enlargement (β*year = 0.030, 95% CI = 0.014–0.045), and more pronounced volume loss in the hippocampus (β*year = −0.068, 95% CI = −0.095 to −0.041) and in the amygdala (β*year = −0.070, 95% CI = −0.109 to −0.031) compared with those with low NfL levels. Analyses using absolute—instead of standardized—brain volumes are reported in Supplementary Table S3.

When considering MTA score, we observed a pattern of association similar to that seen for hippocampal volume, with a significantly faster increase over time among participants with high p‐tau217, NfL, and GFAP compared with those with low biomarker levels (Supplementary Fig S4).

The results remained consistent after further adjustment for *APOE ε*4 carrier status; only the cross‐sectional association between higher GFAP levels and smaller hippocampal volume became marginally significant (Supplementary Table S4). Similar results were observed in sensitivity analyses modeling biomarkers as continuous variables, with all longitudinal associations confirmed, whereas the baseline associations between higher GFAP levels and both larger ventricular volume and smaller hippocampal volume were no longer significant (Supplementary Table S5).

The associations between higher levels of p‐tau217, NfL, and GFAP and faster changes in brain volumes remained consistent after the exclusion of participants with MCI at baseline (Supplementary Table S6) or of those who developed dementia during the 6‐year MRI follow‐up (Supplementary Table S7).

### Associations Between AD‐Related Blood Biomarkers and WMHs Volume

As shown in Figure 2 and Supplementary Table S8, only elevated NfL was associated with faster accumulation of WMHs over time (β*year = 0.033, 95% CI = 0.002–0.063). No associations were observed between the other biomarkers and WMHs either at baseline or during follow‐up. Analyses using absolute – instead of standardized – WMHs volume are reported in Supplementary Table S9.

**FIGURE 2:**
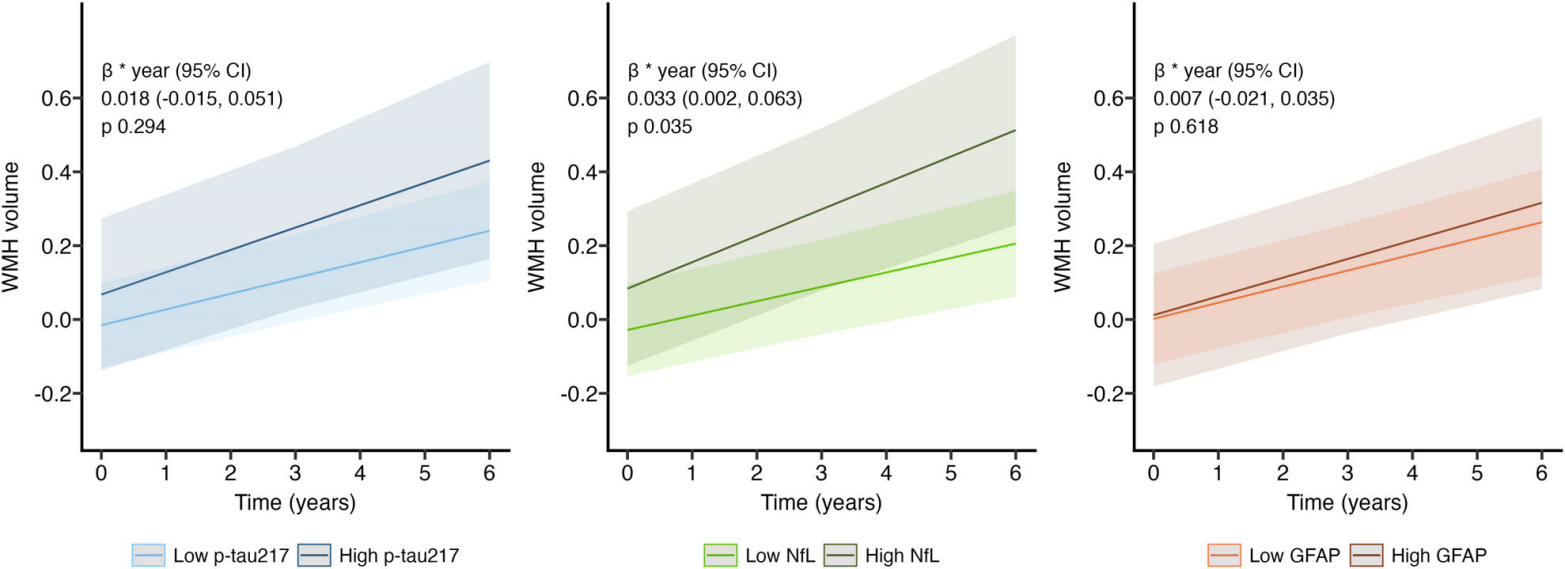
Annual rate of change in WMH volume by baseline levels of p‐tau217, NfL, and GFAP. Linear mixed models adjusted for age, sex, education, chronic kidney disease, anemia, heart disease, and BMI. WMH volume was adjusted for total intracranial volume. Blood biomarkers were dichotomized (high/low) using previously derived optimal cutoffs for 10‐year all‐cause dementia prediction. Cutoffs: 0.134 pg/ml for p‐tau217, 20.171 pg/ml for NfL, and 142.515 pg/ml for GFAP. Number of participants: 357 at baseline, 46 at the 3‐year follow‐up, and 184 at the 6‐year follow‐up. BMI = body mass index; GFAP = glial fibrillary acidic protein; NfL = neurofilament light chain; p‐tau217 = phosphorylated tau 217; WMH = white matter hyperintensity.

The results remained consistent after further adjustment for *APOE ε*4 carrier status (Supplementary Table S10). In sensitivity analyses modeling biomarkers as continuous variables, a cross‐sectional association also emerged between higher NfL levels and larger WMHs volume at baseline (Supplementary Table S11).

After excluding participants with MCI at baseline, the association between NfL and faster WMH accumulation was attenuated and no longer statistically significant, although the point estimate remained similar (Supplementary Table S12), whereas it remained significant after excluding those who developed dementia during the 6‐year MRI follow‐up (Supplementary Table S13).

## Discussion

In cognitively unimpaired community‐dwelling older adults, AD‐related blood biomarkers were associated with distinct patterns of structural brain changes. Elevated levels of p‐tau217 were associated with a faster shrinkage in examined AD‐specific brain regions, namely the hippocampus and amygdala, as well as with a steeper increase in MTA score, but not with total brain volume loss or ventricular enlargement. In contrast, higher concentrations of GFAP and NfL were linked to more diffuse brain alterations, including faster TBTV loss and enlargement of the lateral ventricles, alongside steeper hippocampal and amygdala (NfL) volume loss. Furthermore, NfL was the only biomarker associated with faster accumulation of vascular pathology over time, as reflected by an increasing load of WMH volume. Notably, these associations were observed in a population without evidence of clinical dementia, neurological disorders, or prior cerebrovascular events, and remained consistent after the exclusion of participants with MCI at baseline. This aligns with current models describing the temporal evolution of AD biomarkers, which posit that changes in fluid biomarkers and MRI measures precede the clinical onset of dementia by several years.^33^ Together, our findings thus add to the accumulating evidence that elevated AD‐related blood biomarker can detect subtle brain changes from the earliest disease stage and extends this evidence to a general population of older adults living in the community.

Most existing evidence on the relationship between AD‐related blood biomarkers and structural brain alterations derives from cross‐sectional studies and from research conducted in clinical cohorts, primarily ADNI and BioFINDER.^12^ By contrast, longitudinal studies in population‐based cohorts of older adults free from dementia are few and have yielded mixed results. In line with our findings, a study from the UK Biobank by Huang et al^34^ reported that elevated plasma GFAP and NfL at baseline were associated with faster decline in several cortical and subcortical regions, including fusiform cortex, superior frontal gyrus, pars opercularis, and hippocampus, whereas high plasma p‐tau181 predicted atrophy mainly in the hippocampus. Conversely, findings from the Baltimore Longitudinal Study of Aging^35^ showed that higher p‐tau181 levels were associated with faster grey matter decline in multiple temporal regions and the amygdala, whereas GFAP was primarily linked to longitudinal ventricular enlargement, and no robust associations were observed for NfL. Importantly, neither of these studies included p‐tau217, a biomarker of particular relevance as it is currently the most accurate blood biomarker of AD pathology^20^, ^36^, ^37^ and the only one included as a potential Core 1 biomarker in the revised criteria for the diagnosis and staging of AD.^2^ In addition, some studies suggest that p‐tau217 may become abnormal earlier than p‐tau181 across the AD continuum,^38^ potentially reflecting very early AD‐related pathological changes. Consistent with this framework, our findings identify p‐tau217 as the biomarker most specifically related to longitudinal atrophy in AD‐related brain regions.

In AD, neurofibrillary tangles initially appear in the medial temporal lobe, preceding the onset of clinical symptoms,^39^ and subsequently spread to adjacent temporal and parietal areas, with brain atrophy typically following a similar trajectory.^25^, ^39^, ^40^ The faster decline in hippocampal and amygdala volumes, together with the steeper increase in MTA score, that we observed in participants with high p‐tau217 levels appear consistent with this well‐established pathological progression. Our findings further align with results from the BioFINDER‐2 study,^41^ which reported that elevated p‐tau217 levels predicted temporal cortical thinning and faster hippocampal atrophy in individuals without dementia referred to a memory clinic. Detecting these associations in a population‐based, cognitively and neurologically unimpaired cohort, reinforces the role of p‐tau217 as a sensitive marker of early AD‐related neuronal loss.

Whereas p‐tau217 showed clearly larger effect estimates for volume loss in AD‐related regions compared with global brain volume, NfL and GFAP were associated with more widespread structural brain changes, including overall brain tissue loss and ventricular enlargement. This more diffuse pattern is consistent with the broader nature of these markers: NfL reflects unspecific neuronal loss, whereas GFAP captures astroglial activation, and neither of these processes is unique to AD.^42^, ^43^, ^44^ Accordingly, elevated levels of both biomarkers have been linked to structural brain changes not only in AD,^45^ but also in other neurodegenerative disorders, such as Lewy body disease and frontotemporal lobar degeneration,^46^, ^47^ as well as in non‐neurodegenerative conditions as, for example, cerebrovascular and neuroinflammatory diseases.^44^, ^48^ In particular, NfL has been repeatedly linked to cerebrovascular pathology,^42^ in line with our observation that higher NfL levels were longitudinally associated with faster WMH accumulation. WMH, a core neuroimaging marker of small vessel disease, is commonly observed in older adults and represent a key contributor to cognitive decline.^29^, ^49^ Together, our findings suggest that even in individuals without clinically overt cerebrovascular disease, higher NfL levels may reflect ongoing vascular pathology, underscoring its potential role as a biomarker of overall brain health, sensitive to mixed neuropathological mechanisms. Future studies using higher‐resolution MRI and including a broader range of cerebral small vessel disease markers will be essential to elucidate the association between NfL and cerebrovascular pathology more comprehensively in the community.

In our study, most associations emerged longitudinally rather than at baseline, whereas previous cross‐sectional studies reported associations between AD‐related blood biomarkers and structural MRI measures.^16^, ^47^, ^50^ This difference is likely due to our selection of a very healthy sample drawn from the general population, excluding participants with dementia, cerebrovascular events, or other neurological diseases, which probably resulted in limited baseline variability in brain structure. In such healthy older adult cohorts, the absence of cross‐sectional associations may reflect selective survival bias, whereby individuals with higher biomarker levels and more advanced brain pathology are less likely to remain eligible for inclusion at baseline. In comparison, previous studies typically involved more heterogeneous populations, including individuals with dementia, who presumably exhibited more pronounced brain alterations.

The major strengths of this study include its population‐based design, the longitudinal data for brain MRI, and the assessment of a comprehensive panel of AD‐related blood biomarkers. To the best of our knowledge, this is the first longitudinal population‐based study exploring the link between AD‐related blood biomarkers and longitudinal MRI measures to include p‐tau217. Moreover, SNAC‐K participants underwent a detailed clinical and lifestyle assessment, which enabled careful adjustment for several potential confounders known to influence biomarker concentrations.^32^, ^51^ Our study has also some limitations. First, MRI data were acquired at 1.5T, which, although widely used in routine clinical practice, may limit the sensitivity to detect subtle structural and cerebrovascular changes. Second, structural MRI markers were derived using a combination of automated and manual approaches, which could affect reproducibility across cohorts. Additionally, our analyses focused primarily on global and medial temporal lobe measures, without examining other regional volumes in detail. Similarly, cerebrovascular burden was assessed using WMH volume only. Although WMHs are considered a core neuroimaging marker of cerebral small vessel disease, they do not capture its full spectrum, and other manifestations (eg, microbleeds) were not assessed in our study. Finally, the biomarkers were measured in serum rather than plasma, which may result in lower concentrations, although previous studies have shown strong correlations and comparable diagnostic accuracy between the two matrices.^52^, ^53^

## Conclusions

Based on our findings, cognitively unimpaired older adults with high levels of AD‐related blood biomarkers exhibited a faster progression of structural brain changes over time. The examined biomarkers displayed differential associations with studied longitudinal brain alterations. Elevated p‐tau217 was linked to a faster decline in AD‐related regions, namely the hippocampus and amygdala. In contrast, elevated concentrations of GFAP and NfL were associated with more diffuse alterations, including global brain tissue loss and ventricular enlargement, consistent with their roles as broader markers of neuronal injury and glial responses. Notably, NfL was also associated with greater cerebrovascular burden, underscoring its sensitivity to mixed neuropathological mechanisms. Taken together, these findings indicate that AD‐related blood biomarkers can detect faster progression of neurodegenerative and vascular brain alterations even in the absence of clinical symptoms. This has important implications for both research and clinical practice, suggesting that distinct biomarker profiles may help disentangle early disease‐related processes, refine risk stratification in the community, and ultimately inform the timing and targeting of preventive or disease‐modifying interventions.

## Author Contributions

M.V. and G.G. contributed to the conception and design of the study. M.V., C.F., G.K., and J.O. contributed to the acquisition and analysis of data. M.V., D.L.V., E.J.L., D.R., M.C., C.Q., B.W., L.F., and G.G. contributed to drafting the text or preparing the figures.

## Potential Conflicts of Interest

The authors declare no conflict of interest.

## Supporting information


**Supplementary Figure S1.** Flow chart of study participation.
**Supplementary Figure S2.** Individual trajectories of brain volumes, stratified by baseline levels of p‐tau217, NfL, and GFAP.
**Supplementary Figure S3.** Individual trajectories of white matter hyperintensities (WMH) volumes, stratified by baseline levels of p‐tau217, NfL, and GFAP.
**Supplementary Figure S4.** Annual rate of change in medial temporal atrophy (MTA) score by baseline levels of p‐tau217, NfL, and GFAP.
**Supplementary Table S1.** Cross‐classification of high and low p‐tau217, NfL, and GFAP levels in the baseline study population.
**Supplementary Table S2.** Association of baseline levels of Alzheimer's disease (AD)‐related blood biomarkers with baseline levels and annual rate of change in standardized brain volumes.
**Supplementary Table S3.** Association of baseline levels of Alzheimer's disease (AD)‐related blood biomarkers with baseline levels and annual rate of change in absolute brain volumes.
**Supplementary Table S4.** Association of baseline levels of Alzheimer's disease (AD)‐related blood biomarkers with baseline levels and annual rate of change in standardized brain volumes, adjusted for *APOE‐ε4* carrier status.
**Supplementary Table S5.** Association of baseline continuous levels of Alzheimer's disease (AD)‐related blood biomarkers with baseline levels and annual rate of change in standardized brain volumes.
**Supplementary Table S6.** Association of baseline levels of Alzheimer's disease (AD)‐related blood biomarkers with baseline levels and annual rate of change in standardized brain volumes, excluding 68 participants with MCI at baseline.
**Supplementary Table S7.** Association of baseline levels of Alzheimer's disease (AD)‐related blood biomarkers with baseline levels and annual rate of change in standardized brain volumes, excluding 13 participants who developed dementia during the 6‐years MRI follow‐up.
**Supplementary Table S8.** Association of baseline levels of Alzheimer's disease (AD)‐related blood biomarkers with baseline levels and annual rate of change in standardized white matter hyperintensities volume.
**Supplementary Table S9.** Association of baseline levels of Alzheimer's disease (AD)‐related blood biomarkers with baseline levels and annual rate of change in absolute white matter hyperintensities volume.
**Supplementary Table S10.** Association of baseline levels of Alzheimer's disease (AD)‐related blood biomarkers with baseline levels and annual rate of change in standardized white matter hyperintensities volume, adjusted for *APOE‐ε4* carrier status.
**Supplementary Table S11.** Association of baseline continuous levels of Alzheimer's disease (AD)‐related blood biomarkers with baseline levels and annual rate of change in standardized white matter hyperintensities volume.
**Supplementary Table S12.** Association of baseline levels of Alzheimer's disease (AD)‐related blood biomarkers with baseline levels and annual rate of change in standardized white matter hyperintensities volume, excluding 68 participants with MCI at baseline.
**Supplementary Table S13.** Association of baseline levels of Alzheimer's disease (AD)‐related blood biomarkers with baseline levels and annual rate of change in standardized white matter hyperintensities volume, excluding 13 participants who developed dementia during the 6‐years MRI follow‐up.

## Data Availability

SNAC‐K data can be requested by qualified researchers at https://www.snac-k.se/.
