# Supplementary material for: Blood Biomarkers of Alzheimer's Disease and Patterns of Structural Brain Changes in the Community
Source: Ann Neurol. 2026 Jul 27;100(5):987–97. doi: 10.1002/ana.78309 (PMC13688069; doi:10.1002/ana.78309)
Supplement: Supplementary file 1 [file ANA-100-987-s001.docx]

**Supplementary Material**

**Supplementary Methods. Magnetic resonance imaging (MRI) data acquisition**

All participants underwent 1.5T MRI scans (Philips Intera, The Netherlands) using the same scanner and sequences at baseline and follow-up.

The protocol included a 3D axial magnetization-prepared rapid gradient-echo (MP-RAGE) T1-weighted sequence (resolution: 0.94 × 0.94 × 1.5 mm; no gap; repetition time, TR = 15 ms; echo-time, TE = 7 ms; flip angle, FA = 15°), a proton-density/T2-weighted sequence (resolution: 0.98 × 0.98 × 3 mm; no gap; TR = 3,995 ms; TE = 18/90 ms; echo-train length, ETL = 6; FA = 90°), and a fluid-attenuated inversion recovery (FLAIR) sequence (resolution: 0.90 × 0.90 × 6 mm; gap between slices: 1 mm; TR = 6,000 ms; TE = 100 ms; inversion time, TI = 1900 ms; ETL = 21; FA = 90°).

**Supplementary Figure 1**. Flow chart of study participation

**Supplementary Figure 2**. Individual trajectories of brain volumes, stratified by baseline levels of p-tau217, NfL and GFAP.

Abbreviations: GFAP: glial fibrillary acidic protein; NfL: neurofilament light chain; p-tau217: phosphorylated tau 217; TBTV: total brain tissue volume.

**Supplementary Figure 3**. Individual trajectories of white matter hyperintensities (WMH) volumes, stratified by baseline levels of p-tau217, NfL and GFAP.

Abbreviations: GFAP: glial fibrillary acidic protein; NfL: neurofilament light chain; p-tau217: phosphorylated tau 217; WMH: white matter hyperintensities.

**Supplementary Figure 4**. Annual rate of change in medial temporal atrophy (MTA) score by baseline levels of p-tau217, NfL and GFAP.

Linear mixed models adjusted for age, sex, education, chronic kidney disease, anemia, heart disease and body mass index (BMI). Blood biomarkers were dichotomized (high/low) using previously derived optimal cut-offs for 10-year all-cause dementia prediction. Cut-offs: 0.134 pg/mL for p-tau217, 20.171 pg/mL for NfL and 142.515 pg/mL for GFAP. Abbreviations: GFAP: glial fibrillary acidic protein; MTA: medial temporal atrophy; NfL: neurofilament light chain; p-tau217: phosphorylated tau 217.

**Supplementary Table 1**. Cross-classification of high and low p-tau217, NfL, and GFAP levels in the baseline study population.

| **p-tau217** | **NfL** | **GFAP** | **N (%)** |
| --- | --- | --- | --- |
| High | High | High | 36 (10%) |
| High | High | Low | 17 (4.7%) |
| High | Low | High | 10 (2.8%) |
| High | Low | Low | 29 (8%) |
| Low | High | High | 29 (8%) |
| Low | High | Low | 24 (6.6%) |
| Low | Low | High | 36 (10%) |
| Low | Low | Low | 180 (49.9%) |

Blood biomarkers were dichotomized (high/low) using previously derived optimal cut-offs for 10-year all-cause dementia prediction. Cut-offs: 0.134 pg/mL for p-tau217, 20.171 pg/mL for NfL and 142.515 pg/mL for GFAP. Abbreviations: GFAP: glial fibrillary acidic protein; NfL: neurofilament light chain; p-tau217: phosphorylated tau 217.

**Supplementary Table 2**. Association of baseline levels of Alzheimer’s disease (AD)-related blood biomarkers with baseline levels and annual rate of change in standardized brain volumes.

|  | **TBTV**  **(z-score)** | **Ventricular volume**  **(z-score)** | **Hippocampus volume**  **(z-score)** | **Amygdala volume**  **(z-score)** |
| --- | --- | --- | --- | --- |
| **Baseline** | β (95% CI) | β (95% CI) | β (95% CI) | β (95% CI) |
| **P-tau217** High vs Low | 0.089 (-0.080, 0.258) | 0.155 (-0.058, 0.368) | 0.042 (-0.156, 0.240) | -0.045 (-0.276, 0.186) |
| **NfL** High vs Low | 0.023 (-0.160, 0.206) | 0.113 (-0.119, 0.346) | -0.043 (-0.255, 0.170) | 0.020 (-0.230, 0.270) |
| **GFAP** High vs Low | -0.051 (-0.219, 0.117) | **0.253 (0.042, 0.464)** | **-0.196 (-0.392, 0.000)** | -0.097 (-0.328, 0.134) |
| **Longitudinal** | β*year (95% CI) | β*year (95% CI) | β*year (95% CI) | β*year (95% CI) |
| **P-tau217** High vs Low | -0.013 (-0.036, 0.010) | 0.015 (-0.002, 0.033) | **-0.047 (-0.077, -0.018)** | **-0.064 (-0.105, -0.022)** |
| **NfL** High vs Low | **-0.053 (-0.074, -0.033)** | **0.030 (0.014, 0.045)** | **-0.068 (-0.095, -0.041)** | **-0.070 (-0.109, -0.031)** |
| **GFAP** High vs Low | **-0.040 (-0.059, -0.021)** | **0.028 (0.013, 0.042)** | **-0.038 (-0.064, -0.013)** | -0.024 (-0.060, 0.013) |

Linear mixed models adjusted for age, sex, education, chronic kidney disease, anemia, heart disease and body mass index (BMI). All volumes were adjusted for total intracranial volume. Blood biomarkers were dichotomized (high/low) using previously derived optimal cut-offs for 10-year all-cause dementia prediction. Cut-offs: 0.134 pg/mL for p-tau217, 20.171 pg/mL for NfL and 142.515 pg/mL for GFAP. Abbreviations: GFAP: glial fibrillary acidic protein; NfL: neurofilament light chain; p-tau217: phosphorylated tau 217; TBTV: total brain tissue volume.

**Supplementary Table 3**. Association of baseline levels of Alzheimer’s disease (AD)-related blood biomarkers with baseline levels and annual rate of change in absolute brain volumes.

|  | **TBTV**  **(ml)** | **Ventricular volume**  **(ml)** | **Hippocampus volume**  **(ml)** | **Amygdala volume**  **(ml)** |
| --- | --- | --- | --- | --- |
| **Baseline** | β (95% CI) | β (95% CI) | β (95% CI) | β (95% CI) |
| **P-tau217** High vs Low | 6.56 (-5.83, 18.95) | 2.61 (-0.97, 6.20) | 0.03 (-0.13, 0.20) | -0.01 (-0.09, 0.06) |
| **NfL** High vs Low | 1.71 (-11.76, 15.17) | 1.68 (-2.26, 5.61) | -0.03 (-0.21, 0.15) | 0.01 (-0.07, 0.08) |
| **GFAP** High vs Low | -3.67 (-15.98, 8.63) | **4.25 (0.70, 7.81)** | **-0.16 (-0.32, 0.00)** | -0.03 (-0.11, 0.04) |
| **Longitudinal** | β*year (95% CI) | β*year (95% CI) | β*year (95% CI) | β*year (95% CI) |
| **P-tau217** High vs Low | -0.98 (-2.67, 0.71) | 0.26 (-0.04, 0.56) | **-0.04 (-0.06, -0.01)** | **-0.02 (-0.03, -0.01)** |
| **NfL** High vs Low | **-3.93 (-5.44, -2.41)** | **0.50 (0.23, 0.76)** | **-0.06 (-0.08, -0.03)** | **-0.02 (-0.03, -0.01)** |
| **GFAP** High vs Low | **-2.96 (-4.34, -1.58)** | **0.47 (0.23, 0.71)** | **-0.03 (-0.05, -0.01)** | -0.01 (-0.02, 0.01) |

Linear mixed models adjusted for age, sex, education, chronic kidney disease, anemia, heart disease and body mass index (BMI). All volumes were adjusted for total intracranial volume. Blood biomarkers were dichotomized (high/low) using previously derived optimal cut-offs for 10-year all-cause dementia prediction. Cut-offs: 0.134 pg/mL for p-tau217, 20.171 pg/mL for NfL and 142.515 pg/mL for GFAP. Abbreviations: GFAP: glial fibrillary acidic protein; NfL: neurofilament light chain; p-tau217: phosphorylated tau 217; TBTV: total brain tissue volume.

**Supplementary Table 4**. Association of baseline levels of Alzheimer’s disease (AD)-related blood biomarkers with baseline levels and annual rate of change in standardized brain volumes, adjusted for *APOE-ε4* carrier status.

|  | **TBTV**  **(z-score)** | **Ventricular volume**  **(z-score)** | **Hippocampus volume**  **(z-score)** | **Amygdala volume**  **(z-score)** |
| --- | --- | --- | --- | --- |
| **Baseline** | β (95% CI) | β (95% CI) | β (95% CI) | β (95% CI) |
| **P-tau217** High vs Low | 0.119 (-0.049, 0.288) | 0.158 (-0.058, 0.373) | 0.082 (-0.119, 0.284) | 0.010 (-0.222, 0.242) |
| **NfL** High vs Low | 0.051 (-0.133, 0.234) | 0.064 (-0.174, 0.301) | -0.033 (-0.251, 0.185) | 0.033 (-0.218, 0.284) |
| **GFAP** High vs Low | -0.046 (-0.213, 0.120) | **0.276 (0.063, 0.489)** | -0.198 (-0.396, 0.001) | -0.084 (-0.315, 0.146) |
| **Longitudinal** | β*year (95% CI) | β*year (95% CI) | β*year (95% CI) | β*year (95% CI) |
| **P-tau217** High vs Low | -0.014 (-0.037, 0.009) | 0.015 (-0.002, 0.033) | **-0.048 (-0.078, -0.019)** | **-0.065 (-0.107, -0.024)** |
| **NfL** High vs Low | **-0.054 (-0.074, -0.033)** | **0.030 (0.014, 0.046)** | **-0.068 (-0.095, -0.041)** | **-0.070 (-0.109, -0.031)** |
| **GFAP** High vs Low | **-0.040 (-0.059, -0.021)** | **0.028 (0.013, 0.042)** | **-0.038 (-0.063, -0.013)** | -0.024 (-0.061, 0.012) |

Linear mixed models adjusted for age, sex, education, chronic kidney disease, anemia, heart disease, body mass index (BMI) and apolipoprotein-E (*APOE*) ε4 carrier status. All volumes were adjusted for total intracranial volume. Blood biomarkers were dichotomized (high/low) using previously derived optimal cut-offs for 10-year all-cause dementia prediction. Cut-offs: 0.134 pg/mL for p-tau217, 20.171 pg/mL for NfL and 142.515 pg/mL for GFAP. Abbreviations: GFAP: glial fibrillary acidic protein; NfL: neurofilament light chain; p-tau217: phosphorylated tau 217; TBTV: total brain tissue volume.

**Supplementary Table 5**. Association of baseline continuous levels of Alzheimer’s disease (AD)-related blood biomarkers with baseline levels and annual rate of change in standardized brain volumes.

|  | **TBTV**  **(z-score)** | **Ventricular volume**  **(z-score)** | **Hippocampus volume**  **(z-score)** | **Amygdala volume**  **(z-score)** |
| --- | --- | --- | --- | --- |
| **Baseline** | β (95% CI) | β (95% CI) | β (95% CI) | β (95% CI) |
| **P-tau217** | 0.085 (-0.071, 0.242) | -0.007 (-0.205, 0.192) | 0.135 (-0.048, 0.318) | -0.062 (-0.275, 0.152) |
| **NfL** | -0.079 (-0.213, 0.054) | 0.092 (-0.078, 0.262) | -0.076 (-0.232, 0.079) | -0.014 (-0.196, 0.169) |
| **GFAP** | -0.040 (-0.094, 0.014) | 0.036 (-0.032, 0.105) | -0.051 (-0.114, 0.012) | -0.028 (-0.101, 0.046) |
| **Longitudinal** | β*year (95% CI) | β*year (95% CI) | β*year (95% CI) | β*year (95% CI) |
| **P-tau217** | -0.012 (-0.032, 0.008) | 0.014 (-0.001, 0.030) | **-0.037 (-0.063, -0.012)** | **-0.043 (-0.079, -0.006)** |
| **NfL** | **-0.065 (-0.092, -0.038)** | **0.039 (0.019, 0.060)** | **-0.090 (-0.124, -0.055)** | **-0.073 (-0.123, -0.022)** |
| **GFAP** | **-0.028 (-0.045, -0.010)** | **0.015 (0.003, 0.026)** | **-0.031 (-0.053, -0.008)** | -0.027 (-0.058, 0.004) |

Linear mixed models adjusted for age, sex, education, chronic kidney disease, anemia, heart disease and body mass index (BMI). All volumes were adjusted for total intracranial volume. Blood biomarkers were standardized into z-scores. Abbreviations: GFAP: glial fibrillary acidic protein; NfL: neurofilament light chain; p-tau217: phosphorylated tau 217; TBTV: total brain tissue volume.

**Supplementary Table 6**. Association of baseline levels of Alzheimer’s disease (AD)-related blood biomarkers with baseline levels and annual rate of change in standardized brain volumes, excluding 68 participants with MCI at baseline.

|  | **TBTV**  **(z-score)** | **Ventricular volume**  **(z-score)** | **Hippocampus volume**  **(z-score)** | **Amygdala volume**  **(z-score)** |
| --- | --- | --- | --- | --- |
| **Baseline** | β (95% CI) | β (95% CI) | β (95% CI) | β (95% CI) |
| **P-tau217** High vs Low | **0.195 (0.015, 0.376)** | 0.086 (-0.146, 0.317) | 0.118 (-0.098, 0.334) | 0.018 (-0.227, 0.263) |
| **NfL** High vs Low | 0.087 (-0.114, 0.288) | 0.088 (-0.171, 0.348) | 0.035 (-0.203, 0.274) | 0.142 (-0.129, 0.413) |
| **GFAP** High vs Low | -0.108 (-0.292, 0.075) | **0.289 (0.055, 0.523)** | -0.213 (-0.432, 0.005) | -0.118 (-0.368, 0.132) |
| **Longitudinal** |  |  |  |  |
| **P-tau217** High vs Low | -0.012 (-0.037, 0.012) | 0.012 (-0.007, 0.031) | **-0.046 (-0.079, -0.013)** | **-0.055 (-0.101, -0.009)** |
| **NfL** High vs Low | **-0.060 (-0.082, -0.039)** | **0.038 (0.021, 0.054)** | **-0.071 (-0.101, -0.041)** | **-0.085 (-0.127, -0.044)** |
| **GFAP** High vs Low | **-0.046 (-0.066, -0.026)** | **0.033 (0.018, 0.049)** | **-0.045 (-0.074, -0.016)** | -0.024 (-0.065, 0.017) |

Linear mixed models adjusted for age, sex, education, chronic kidney disease, anemia, heart disease and body mass index (BMI). All volumes were adjusted for total intracranial volume. Blood biomarkers were dichotomized (high/low) using previously derived optimal cut-offs for 10-year all-cause dementia prediction. Cut-offs: 0.134 pg/mL for p-tau217, 20.171 pg/mL for NfL and 142.515 pg/mL for GFAP. Abbreviations: GFAP: glial fibrillary acidic protein; NfL: neurofilament light chain; p-tau217: phosphorylated tau 217; TBTV: total brain tissue volume.

**Supplementary Table 7**. Association of baseline levels of Alzheimer’s disease (AD)-related blood biomarkers with baseline levels and annual rate of change in standardized brain volumes, excluding 13 participants who developed dementia during the six-years MRI follow-up.

|  | **TBTV**  **(z-score)** | **Ventricular volume**  **(z-score)** | **Hippocampus volume**  **(z-score)** | **Amygdala volume**  **(z-score)** |
| --- | --- | --- | --- | --- |
| **Baseline** | β (95% CI) | β (95% CI) | β (95% CI) | β (95% CI) |
| **P-tau217** High vs Low | 0.074 (-0.102, 0.251) | 0.125 (-0.089, 0.339) | 0.062 (-0.140, 0.265) | -0.009 (-0.247, 0.228) |
| **NfL** High vs Low | 0.010 (-0.177, 0.198) | 0.070 (-0.160, 0.299) | -0.037 (-0.251, 0.177) | -0.018 (-0.269, 0.233) |
| **GFAP** High vs Low | -0.051 (-0.223, 0.121) | **0.218 (0.009, 0.427)** | **-0.203 (-0.400, -0.006)** | -0.124 (-0.357, 0.108) |
| **Longitudinal** | β*year (95% CI) | β*year (95% CI) | β*year (95% CI) | β*year (95% CI) |
| **P-tau217** High vs Low | -0.011 (-0.034, 0.012) | 0.015 (-0.003, 0.033) | **-0.044 (-0.074, -0.015)** | **-0.063 (-0.105, -0.021)** |
| **NfL** High vs Low | **-0.052 (-0.072, -0.031)** | **0.030 (0.014, 0.046)** | **-0.066 (-0.093, -0.039)** | **-0.069 (-0.108, -0.030)** |
| **GFAP** High vs Low | **-0.039 (-0.058, -0.020)** | **0.028 (0.014, 0.042)** | **-0.037 (-0.062, -0.011)** | -0.023 (-0.059, 0.014) |

Linear mixed models adjusted for age, sex, education, chronic kidney disease, anemia, heart disease and body mass index (BMI). All volumes were adjusted for total intracranial volume. Blood biomarkers were dichotomized (high/low) using previously derived optimal cut-offs for 10-year all-cause dementia prediction. Cut-offs: 0.134 pg/mL for p-tau217, 20.171 pg/mL for NfL and 142.515 pg/mL for GFAP. Abbreviations: GFAP: glial fibrillary acidic protein; NfL: neurofilament light chain; p-tau217: phosphorylated tau 217; TBTV: total brain tissue volume.

**Supplementary Table 8**. Association of baseline levels of Alzheimer’s disease (AD)-related blood biomarkers with baseline levels and annual rate of change in standardized white matter hyperintensities volume.

|  | **White Matter Hyperintensities Volume (z-score)** | |
| --- | --- | --- |
|  | Baseline  β (95% CI) | Longitudinal  β*year (95% CI) |
| **P-tau217** High vs Low | 0.083 (-0.156, 0.322) | 0.018 (-0.015, 0.051) |
| **NfL** High vs Low | 0.122 (-0.138, 0.381) | **0.033 (0.002, 0.063)** |
| **GFAP** High vs Low | 0.010 (-0.229, 0.250) | 0.007 (-0.021, 0.035) |

Linear mixed models adjusted for age, sex, education, chronic kidney disease, anemia, heart disease and body mass index (BMI). White matter hyperintensities volume was adjusted for total intracranial volume. Blood biomarkers were dichotomized (high/low) using previously derived optimal cut-offs for 10-year all-cause dementia prediction. Cut-offs: 0.134 pg/mL for p-tau217, 20.171 pg/mL for NfL and 142.515 pg/mL for GFAP. Abbreviations: GFAP: glial fibrillary acidic protein; NfL: neurofilament light chain; p-tau217: phosphorylated tau 217.

**Supplementary Table 9**. Association of baseline levels of Alzheimer’s disease (AD)-related blood biomarkers with baseline levels and annual rate of change in absolute white matter hyperintensities volume.

|  | **White Matter Hyperintensities Volume (ml)** | |
| --- | --- | --- |
|  | Baseline  β (95% CI) | Longitudinal  β*year (95% CI) |
| **P-tau217** High vs Low | 0.73 (-1.37, 2.83) | 0.16 (-0.13, 0.45) |
| **NfL** High vs Low | 0.98 (-1.31, 3.27) | **0.29 (0.02, 0.55)** |
| **GFAP** High vs Low | 0.09 (-2.01, 2.19) | 0.06 (-0.18, 0.31) |

Linear mixed models adjusted for age, sex, education, chronic kidney disease, anemia, heart disease and body mass index (BMI). White matter hyperintensities volume was adjusted for total intracranial volume. Blood biomarkers were dichotomized (high/low) using previously derived optimal cut-offs for 10-year all-cause dementia prediction. Cut-offs: 0.134 pg/mL for p-tau217, 20.171 pg/mL for NfL and 142.515 pg/mL for GFAP. Abbreviations: GFAP: glial fibrillary acidic protein; NfL: neurofilament light chain; p-tau217: phosphorylated tau 217.

**Supplementary Table 10**. Association of baseline levels of Alzheimer’s disease (AD)-related blood biomarkers with baseline levels and annual rate of change in standardized white matter hyperintensities volume, adjusted for *APOE-ε4* carrier status.

|  | **White Matter Hyperintensities Volume (z-score)** | |
| --- | --- | --- |
|  | Baseline  β (95% CI) | Longitudinal  β*year (95% CI) |
| **P-tau217** High vs Low | 0.082 (-0.159, 0.323) | 0.018 (-0.016, 0.051) |
| **NfL** High vs Low | 0.052 (-0.213, 0.316) | **0.033 (0.003, 0.063)** |
| **GFAP** High vs Low | 0.000 (-0.241, 0.240) | 0.007 (-0.021, 0.035) |

Linear mixed models adjusted for age, sex, education, chronic kidney disease, anemia, heart disease, body mass index (BMI) and apolipoprotein-E (*APOE*) ε4 carrier status. White matter hyperintensities volume was adjusted for total intracranial volume. Blood biomarkers were dichotomized (high/low) using previously derived optimal cut-offs for 10-year all-cause dementia prediction. Cut-offs: 0.134 pg/mL for p-tau217, 20.171 pg/mL for NfL and 142.515 pg/mL for GFAP. Abbreviations: GFAP: glial fibrillary acidic protein; NfL: neurofilament light chain; p-tau217: phosphorylated tau 217.

**Supplementary Table 11**. Association of baseline continuous levels of Alzheimer’s disease (AD)-related blood biomarkers with baseline levels and annual rate of change in standardized white matter hyperintensities volume.

|  | **White Matter Hyperintensities Volume (z-score)** | |
| --- | --- | --- |
|  | Baseline  β (95% CI) | Longitudinal  β*year (95% CI) |
| **P-tau217** | 0.091 (-0.134, 0.317) | 0.018 (-0.011, 0.047) |
| **NfL** | **0.364 (0.051, 0.676)** | **0.040 (0.003, 0.078)** |
| **GFAP** | 0.096 (-0.059, 0.250) | 0.003 (-0.016, 0.023) |

Linear mixed models adjusted for age, sex, education, chronic kidney disease, anemia, heart disease and body mass index (BMI). White matter hyperintensities volume was adjusted for total intracranial volume. Blood biomarkers were standardized into z-scores. Abbreviations: GFAP: glial fibrillary acidic protein; NfL: neurofilament light chain; p-tau217: phosphorylated tau 217.

**Supplementary Table 12**. Association of baseline levels of Alzheimer’s disease (AD)-related blood biomarkers with baseline levels and annual rate of change in standardized white matter hyperintensities volume, excluding 68 participants with MCI at baseline.

|  | **White Matter Hyperintensities Volume (z-score)** | |
| --- | --- | --- |
|  | Baseline  β (95% CI) | Longitudinal  β*year (95% CI) |
| **P-tau217** High vs Low | -0.010 (-0.242, 0.222) | 0.021 (-0.020, 0.061) |
| **NfL** High vs Low | -0.157 (-0.415, 0.101) | 0.030 (-0.006, 0.067) |
| **GFAP** High vs Low | -0.141 (-0.378, 0.096) | 0.012 (-0.023, 0.046) |

Linear mixed models adjusted for age, sex, education, chronic kidney disease, anemia, heart disease and body mass index (BMI). White matter hyperintensities volume was adjusted for total intracranial volume. Blood biomarkers were dichotomized (high/low) using previously derived optimal cut-offs for 10-year all-cause dementia prediction. Cut-offs: 0.134 pg/mL for p-tau217, 20.171 pg/mL for NfL and 142.515 pg/mL for GFAP. Abbreviations: GFAP: glial fibrillary acidic protein; NfL: neurofilament light chain; p-tau217: phosphorylated tau 217.

**Supplementary Table 13**. Association of baseline levels of Alzheimer’s disease (AD)-related blood biomarkers with baseline levels and annual rate of change in standardized white matter hyperintensities volume, excluding 13 participants who developed dementia during the six-years MRI follow-up.

|  | **White Matter Hyperintensities Volume (z-score)** | |
| --- | --- | --- |
|  | Baseline  β (95% CI) | Longitudinal  β*year (95% CI) |
| **P-tau217** High vs Low | -0.097 (-0.311, 0.116) | 0.019 (-0.014, 0.053) |
| **NfL** High vs Low | 0.060 (-0.167, 0.288) | **0.033 (0.003, 0.064)** |
| **GFAP** High vs Low | -0.042 (-0.252, 0.168) | 0.008 (-0.020, 0.037) |

Linear mixed models adjusted for age, sex, education, chronic kidney disease, anemia, heart disease and body mass index (BMI). White matter hyperintensities volume was adjusted for total intracranial volume. Blood biomarkers were dichotomized (high/low) using previously derived optimal cut-offs for 10-year all-cause dementia prediction. Cut-offs: 0.134 pg/mL for p-tau217, 20.171 pg/mL for NfL and 142.515 pg/mL for GFAP. Abbreviations: GFAP: glial fibrillary acidic protein; NfL: neurofilament light chain; p-tau217: phosphorylated tau 217.
